# Nailfold capillaroscopy in myositis: A case series

**DOI:** 10.1177/2050313X251353297

**Published:** 2025-06-27

**Authors:** Thaisa Cotton, Marie Hudson, Yves Troyanov, Valérie Leclair, Geneviève Gyger

**Affiliations:** 1 Department of Medicine, McGill University, Montreal, QC, Canada; 2 Division of Rheumatology, Jewish General Hospital, Montreal, QC, Canada; 3 Lady Davis Institute for Medical Research, Montreal, QC, Canada; 4 Division of Rheumatology, Department of Medicine, Hôpital du Sacre-Coeur de Montreal, University of Montreal, QC, Canada

**Keywords:** nailfold videocapillaroscopy, idiopathic inflammatory myopathies, dermatomyositis, interstitial lung disease, connective tissue disease

## Abstract

Nailfold capillaroscopy is well established in systemic sclerosis; however, abnormalities (scleroderma or scleroderma-like patterns) are identified in other connective tissue diseases, such as myositis, even in the absence of Raynaud’s phenomenon. Although this may be known within the rheumatology world, myositis cases often present to other subspecialists who may be unfamiliar with the value of capillaroscopy. We present four cases of myositis that initially presented to non-rheumatology subspecialists where capillaroscopy played a role in confirming the diagnosis. Nailfold capillaroscopy is especially useful in cases of isolated interstitial lung disease, atypical and isolated rashes, and to delineate between myositis mimickers, whether pathologic or clinical. It is a noninvasive tool, which is useful early in the work-up of myositis to identify features consistent with a connective tissue disease.

## Introduction

Idiopathic inflammatory myopathies (IIM) are a group of acquired muscle diseases characterized by inflammation and weakness. Historically, IIM were classified as dermatomyositis (DM) or polymyositis (PM) based on criteria developed by Bohan and Peter in the 1970s. However, with the description of new clinical subsets, the ongoing discovery of myositis-specific autoantibodies, and a deeper understanding of muscle histopathology, classification has moved toward the use of integrated clinical, serological, and pathological features to subset IIM patients into more homogeneous groups.^1^ At present, there are four main IIM subsets: DM, immune-mediated necrotizing myositis (IMNM), overlap myositis (which includes anti-synthetase syndrome and scleromyositis, among others), and inclusion body myositis (IBM). Each subset has characteristic autoantibodies and histological patterns. In this framework, PM has become a rare entity, and work is ongoing to further delineate more homogeneous subsets of overlap myositis.^1^

Nailfold capillaroscopy is an in vivo and noninvasive morphological evaluation of the nailfold capillaries. There are several methods of performing capillaroscopy, including a dermatoscope (magnification ×10), stereomicroscope (magnification varying ×10–70), and videocapillaroscope (magnification ×200), the last being the gold standard.^2^ The dermatoscope has been found to be reliable when compared to the videocapillaroscope for normal and overt scleroderma patterns.^3^

The EULAR study group on microcirculation in rheumatic diseases published standardized definitions of nailfold capillary abnormalities in IIM.^4^ These were grouped as (semi)-quantitative parameters, including capillary density, capillary dimension, capillary morphology, and microhemorrhages, and qualitative parameters, including scleroderma (early, active, and late) and scleroderma-like patterns. In their structured review of the literature, they identified the value of nailfold videocapillaroscopy (NVC) in distinguishing not only IIM from healthy controls and systemic sclerosis (SSc) but also between IIM subsets. They also highlighted the correlations between NVC abnormalities in IIM and disease activity, as well as treatment response. However, this remains relatively unknown outside of rheumatologists.

In this article, we present four cases of myositis presenting to various other subspecialists where NVC contributed to diagnosis. These were not consecutive cases, but rather illustrative examples selected for their atypical presentation to demonstrate the role of NVC in such cases.

## NVC image acquisition and scoring

NVC images were acquired using the DS MEDICA Videocap (×200 magnification) after subjects were seated in a room at 22°C–25°C for a minimum of 20 min. A drop of immersion oil was applied to the nailfold to maximize translucency of the keratin layer, and the nailfolds of the second, third, fourth, and fifth fingers of both hands were photographed and scored by a trained rheumatologist (G.G.). Four pictures were taken per digit. The following capillaroscopic variables were identified: (1) capillary density, (2) capillary dimensions, (3) capillary morphology, (4) microhemorrhages, and (5) qualitative capillaroscopic parameters, as defined by Piette et al.^4^

## Case reports

### Case 1

A 38-year-old previously healthy black man presented to dermatology with a 1-month history of diffuse erythrodermic and pruritic rash. He was diagnosed with atopic dermatitis but had incomplete improvement with a cortisone cream, so he was treated with prednisone 50 mg with a taper over 42 days. His rash recurred during the prednisone taper, and he was referred to rheumatology. On rheumatology assessment, he had erythroderma, no muscle weakness, and no pulmonary symptoms.

**Figure 1.**
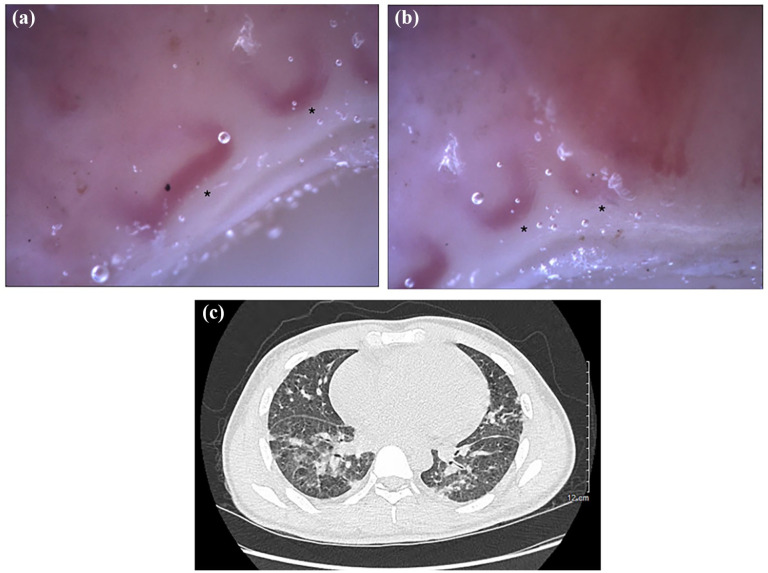
Case 1. (a, b) Panels show the patient’s nailfold videocapillaroscopic pictures at 200× magnification. (a) Panel shows markedly reduced capillary density (one capillary/1 linear mm) and giant capillaries (semi-quantitative score 3). (b) Panel also shows markedly reduced density (two capillaries/1 linear mm) and giant capillaries (semi-quantitative score 3). (c) Panel shows the patient’s CT chest with evidence of interstitial lung disease. *Giant capillaries. CT: computed tomography.

His work-up included a positive antinuclear antibody (ANA, 1:160 cytoplasmic speckled). He had negative extractable-nuclear antigens (ENA), anti-double-stranded-DNA (anti-dsDNA), rheumatoid factor (RF), antineutrophilic cytoplasmic antibodies, and anti-phospholipids. The levels of creatinine kinase (CK, 79 U/L, normal range 42–396 U/L), complements, and C-reactive protein were normal, as well as protein electrophoresis. Radiographs of the hands showed no erosive arthropathy or calcinosis, and a chest radiograph was normal. NVC was performed (Figure 1(a) and (b)) and showed diminished capillary density, giant capillaries, and abnormally shaped capillaries consistent with a scleroderma-like pattern. The patient underwent a skin biopsy, which showed a foci of interface dermatitis favoring an immunopathologic dermatitis rather than eczema. An electromyography (EMG) was normal. However, muscle magnetic resonance imaging was in keeping with myositis in the proximal upper and lower limbs.

He was diagnosed with DM, but he was reluctant to start treatment and lost to follow-up. He presented to the emergency room (ER) several months later with a 1-month history of new dyspnea, non-productive cough, and fevers. A computed tomography (CT) of the thorax revealed new nodular densities bilaterally involving the upper and lower lobes with lymphadenopathy, and a pericardial effusion. (Figure 1(c)). He was admitted to the hospital with the diagnosis of multifocal pneumonia; however, no infectious etiology was identified, and his oxygen requirements increased despite broad-spectrum antibiotic therapy, requiring transfer to the intensive care unit. Pericardial and pleural fluid cytology showed no evidence of infection or malignancy. A wedge lung biopsy indicated diffuse alveolar damage representing the morphological spectrum of acute respiratory distress syndrome with the presence of C3 deposits on immunofluorescence, and no evidence of bacterial, fungal, or viral infection. As there was no clinical improvement despite broad-spectrum antibiotics, antivirals, and antifungals, with no evidence of infection, he was treated as DM with rapidly progressive interstitial lung disease (ILD) with high-dose solumedrol, cyclophosphamide, intravenous immunoglobulins (IVIG), and cyclosporin. Unfortunately, there was no clinical improvement, and he passed away from respiratory failure 2 months after presenting to the ER. His myositis panel resulted post-mortem as positive for anti-melanoma differentiation-associated protein 5 (MDA5). Thus, his case was that of an anti-MDA5 DM with rapidly progressive ILD.

### Case 2

A 54-year-old previously healthy white female presented to dermatology with 2 months of erythroderma covering 95% of her body surface area. She had conflicting skin biopsies with one suggestive of pityriasis rosacea and another raising the suspicion for primary cutaneous T-cell lymphoma. Part of her initial work-up included a positive ANA (1:80 speckled), a negative ENA and RF, and normal complement levels.

**Figure 2.**
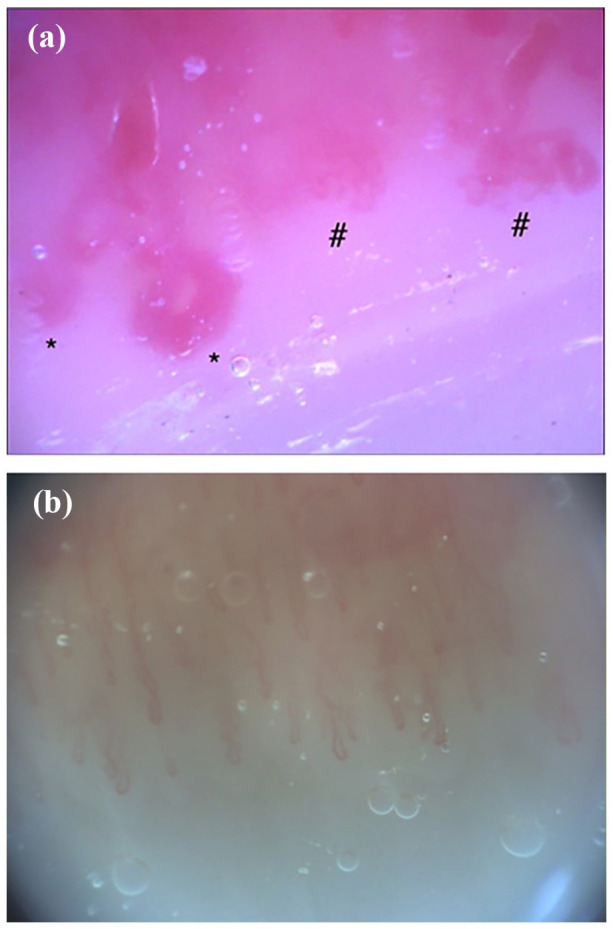
Case 2. (a, b) Panels show the patient’s nailfold videocapillaroscopic pictures at 200× magnification. (a) Panel is her capillaroscopy during active disease, with an abnormal pattern showing reduced density (three capillaries/1 linear mm), giant capillaries (semi-quantitative score 3), and abnormally shaped capillaries (semi-quantitative score 3). (b) Panel shows normalization of her capillaroscopy after achieving disease quiescence, with a normal density (seven capillaries/1 linear mm). *Giant capillaries. ^#^Abnormally shaped capillaries.

She was referred to rheumatology, and on assessment had normal strength and no extra-muscular symptoms. NVC was abnormal, as seen in Figure 2(a), with moderately diminished capillary density, dilated, giant capillaries, and abnormally shaped capillaries consistent with a scleroderma-like pattern. Further work-up revealed normal CK levels (149 U/L, normal range 24–240 U/L), EMG, and muscle biopsy. A CT chest showed no evidence of ILD. A myositis panel resulted positive for anti-small ubiquitin-like modifier-1 activating enzyme (SAE1), so she was diagnosed with anti-SAE1 amyopathic DM. Her skin disease was difficult to treat, failing several treatments due either to intolerance or primary failure, including topical steroids, prednisone, IVIG, hydroxychloroquine, methotrexate, mycophenolate mofetil, and tofacitinib.

Two years following her initial presentation, she developed new proximal weakness with normal CK levels (131 U/L, normal range 24–240 U/L). Her EMG showed fibrillations and sharp waves suggestive of active myositis. She achieved remission with a prednisone taper and rituximab. A follow-up NVC while in remission showed normalization of her capillary abnormalities, as seen in Figure 2(b).

### Case 3

A 78-year-old male from the Philippines, known for a history of prostate cancer in remission, hypertension, type 2 diabetes, dyslipidemia, gout, and chronic kidney disease, presented to the ER with 3 weeks of proximal muscle weakness and plaques on his nasolabial folds and forehead. His home medications included atorvastatin 5 mg daily and no other myotoxic drugs.

**Figure 3.**
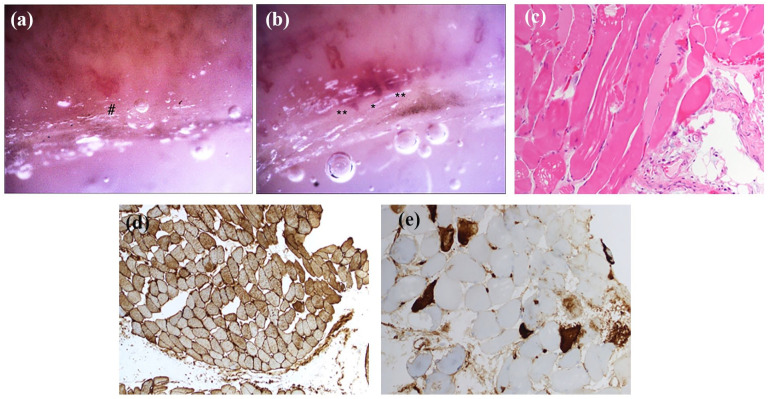
Case 3. (a, b) Panels show the patient’s nailfold videocapillaroscopic pictures at 200× magnification. (a) Panel shows markedly reduced density (one capillary/1 linear mm) and an abnormally shaped capillary (semi-quantitative score 3). (b) Panel shows reduced density (three capillaries/1 linear mm), dilated capillaries (semi-quantitative score 3), and giant capillaries (semi-quantitative score 2). (c–e) Panels show slides of his muscle biopsy. (c) Panel is the H&E stain at 20× magnification showing some scattered necrosis. (d) Panel is the MHC1 stain at 10× magnification, showing strong and diffusely scattered MHC1 stain, with a subtle perimysial predominance. (e) Panel is the MAC stain at 20× magnification, showing MAC positivity in the cytoplasm but also in the capillaries. *Giant capillaries. ^#^Abnormally shaped capillaries. **Dilated capillaries. H&E: hematoxylin and eosin; MAC: membrane attack complex; MHC1: major histocompatibility 1.

His CK level was initially increased at over 24,000 U/L (normal range 42-396 U/L) and stabilized around 21,000 U/L despite several days of aggressive intravenous hydration. NVC was abnormal with moderately to severely diminished capillary density and abnormally shaped capillaries, as seen in Figure 3(a) and (b), suggestive of DM rather than IMNM.^5^ An EMG was also compatible with an inflammatory myositis. His subsequent investigations included a positive ANA (1:640 speckled), negative ENA, and normal complement levels. A CT thorax showed no evidence of ILD. A positron emission tomography showed no evidence of malignancy but had uptake in the proximal muscles consistent with myositis. A muscle biopsy was performed and initially reported as compatible with IMNM, with evidence of some necrosis, diffusely scattered major histocompatibility 1 (MHC1) staining, and cytoplasmic and capillary membrane attack complex staining (Figure 3(c)–(e)). His myositis panel later resulted positive for both anti-Mi2 alpha and anti-Mi2 beta and negative for anti-3-hydroxyl-3-methylglutaryl-coenzyme A reductase. On the revision of his muscle biopsy with this information, subtle perifascicular predominance of strongly positive MHC1 and necrosis was consistent with DM – in keeping with his capillaroscopy and serologies.

### Case 4

**Figure 4.**
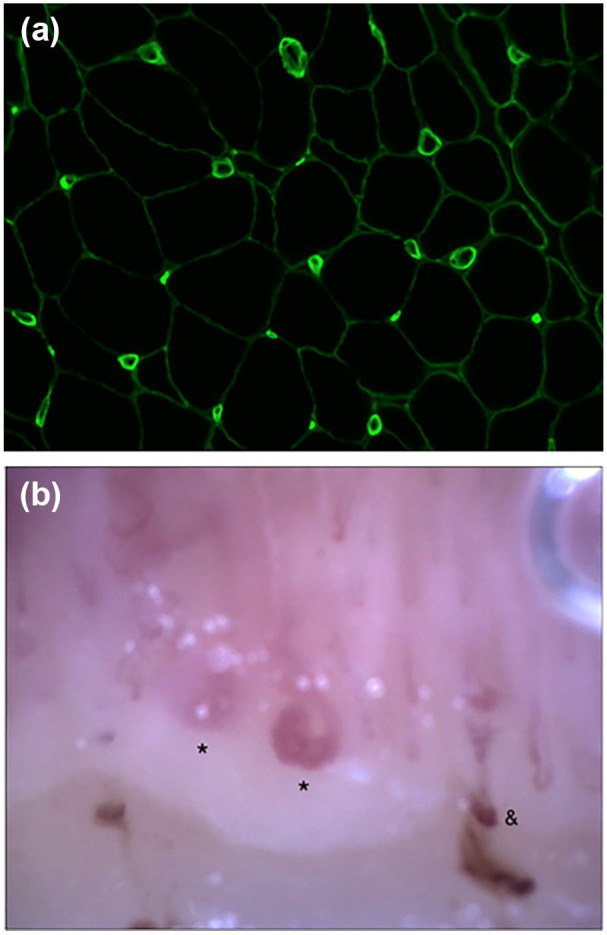
Case 4. (a) Panel shows the collagen 4 stain of the patient’s muscle biopsy at 20× magnification with reduced capillary density and abnormal capillary morphology. (b) Panel shows the patient’s nailfold videocapillaroscopic picture at 200× magnification, with reduced density (six capillaries/1 linear mm), giant capillaries (semi-quantitative score 2), and microhemorrhages. *Giant capillaries. ^&^Microhemorrhages.

A 75-year-old white male known for chronic bronchitis was followed in neurology for asymmetrical proximal and distal weakness, oropharyngeal dysphagia, axial muscle weakness, muscle atrophy, and mildly elevated CK levels up to 628 U/L (normal range 42–396 U/L). Ten years into the disease, he underwent a muscle biopsy to confirm the putative diagnosis of IBM. On the biopsy, the pathologist noted marked capillary abnormalities (Figure 4(a)), including prominent capillary basement membrane reduplication, a finding that has been reported to be a histopathological hallmark of scleromyositis.^6^ Thus, the pathologist recommended a referral to rheumatology.

On rheumatology assessment, the patient had sclerodactyly, finger contractures, digital tip ulcers, Gottron’s papules, gastroesophageal reflux, gastric antral vascular ectasias, and ILD. NVC showed an active scleroderma pattern, as seen in Figure 4(b). Subsequent investigations included a positive ANA (1:1280 nucleolar), and a negative ENA, anti-dsDNA, myositis panel, and scleroderma panel. He was diagnosed with seronegative scleromyositis^7^ and he later improved with methotrexate, IVIG, and mycophenolate mofetil.

## Discussion

**Figure 5.**
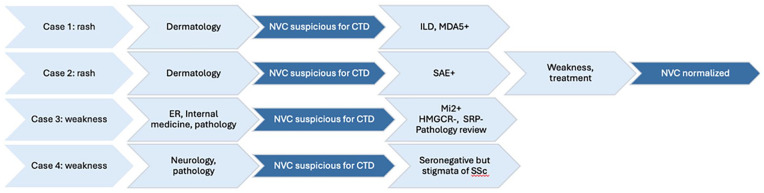
Sequence of events of the four cases (all without Raynaud’s phenomenon) presenting to non-rheumatologists, where NVC raised the suspicion for a connective tissue disease. The sequence is as follows: predominant symptom, initial physician, NVC findings, subsequent serologies, and assessments. CTD: connective tissue disease; ER: emergency room; ILD: interstitial lung disease; NVC: nailfold videocapillaroscopy.

We presented four cases where NVC contributed to the diagnosis of myositis: a patient with anti-MDA5 DM and another with anti-SAE1 DM who initially presented with rashes, a patient with anti-Mi2 DM that mimicked IMNM clinically and pathologically, and a patient with seronegative scleromyositis that mimicked IBM clinically (Figure 5). We specifically selected these four cases to raise awareness of the value of NVC in IIM beyond what is well known to rheumatologists and described in the literature, highlighting atypical presentations when patients may be initially assessed by non-rheumatologists.

In case 1, the patient had a nonspecific rash. In addition, he was black and rashes are known to be more difficult to visualize in people with darker skin tones.^8^ In that setting, abnormal NVC was an important clue pointing towards a possible systemic disease. Unfortunately, he was lost to follow-up, during which time his lung disease progressed. Of note, even in cases of isolated ILD, NVC can be helpful to identify an underlying connective tissue disease early in the work-up. In their prospective study, Sambataro et al. found an abnormal NVC in 18% of their 361 ILD patients, 78% of whom were later diagnosed with a connective tissue disease. Interestingly, 31% of these did not have Raynaud’s phenomenon.^9^ We propose that NVC should be part of the basic work-up of ILD, as it can help clinicians identify early cases of connective tissue diseases.

In case 2, the patient had an atypical rash and multiple conflicting non-diagnostic skin biopsies. Muscle enzymes and serologies were initially normal. Yet NVC was again helpful to raise the suspicion of a possible connective tissue disease. In addition, in this case, disease quiescence was mirrored by normalization of capillaroscopy. In contrast to SSc, normalization or improvement of capillaroscopy with treatment of certain subsets of myositis is a finding that has been noted in the literature, especially in DM.^10–13^ Studies have identified either normalization of capillaroscopy pattern or improvement of capillary density, dimension, and microhemorrhages. Improvement of capillaroscopy abnormalities can be identified as early as 2–17 weeks following the initiation of treatment in anti-MDA5 DM.^13^ Similarly, other studies have also noted a correlation of NVC with disease activity.^12,14^ Although more research is required, NVC may be a useful tool in certain subsets of myositis to follow disease activity.

The predominant feature of case 3 was severe myositis and the muscle biopsy was initially interpreted as IMNM. However, NVC was abnormal and in our experience, this is not a feature of IMNM. Although there are sparse data, to our knowledge, at least one study has found that NVC is normal in IMNM,^15^ and another showed mostly nonspecific changes,^5^ suggesting that abnormal capillaroscopic patterns (i.e. scleroderma or scleroderma-like patterns) may be inconsistent with IMNM. Serologies and careful review of the muscle biopsy were then helpful to confirm the diagnosis. Seeing as there are reports of DM-like rashes in proven IMNM,^16^ routine capillaroscopy performed in all cases of suspected myositis can help delineate between certain IIM subsets. Also, although there have been rare cases of abnormal patterns identified in genetic and metabolic myopathies,^17^ NVC can still be a helpful tool to flag cases of inflammatory myopathies that may require more work-up to rule out an underlying connective tissue disease.

Similarly, NVC helped identify a feature inconsistent with IBM in case 4, ultimately supporting a diagnosis of scleromyositis. Torres-Ruiz et al. reported that IBM had mostly normal capillaroscopy (85.8%) or nonspecific changes (14.2%).^5^ Considering the ongoing discovery of the spectrum of IBM-like phenotypes and mimickers,^18^ which includes scleromyositis, NVC can be another tool for clinicians to delineate between these phenotypes to identify features inconsistent with a pure IBM.

We also consider it important to highlight that none of the cases presented reported Raynaud’s phenomenon. In fact, capillaroscopy abnormalities identified independent of Raynaud’s phenomenon are a finding reflected in the literature for both myositis and connective tissue disease-related ILD.^4,9^ Thus, capillaroscopy can provide useful information as part of the basic work-up of all cases of suspected myositis, even in the absence of Raynaud’s phenomenon.

The EULAR study group on microcirculation in rheumatic diseases published standardized definitions of nailfold capillary abnormalities in IIM.^4^ These include reduced capillary density, dilated and giant capillaries, abnormally shaped capillaries, and microhemorrhages seen in the NVC of the cases presented here. The EULAR study group also describes two main capillary patterns, namely scleroderma (early, active, and late) and scleroderma-like. The first three cases had DM with scleroderma-like patterns, and the fourth had SSc with an active scleroderma pattern. Of note, although the scleroderma pattern is more common in SSc and the scleroderma-like pattern in IIM, these associations are not specific. Indeed, the scleroderma pattern can be seen in IIM,^11^ and the scleroderma-like pattern in SSc.^2^ The terminology may be misleading, and disease-agnostic terminology might be preferable to avoid confusion for non-rheumatologists with less experience in NVC.

This series is not without limitations, including a small sample, potential for selection bias, retrospective data collection, and limited generalizability. Nevertheless, case series still have considerable merit, in this case highlighting the potential value of NVC in challenging cases of myositis that might present to non-rheumatology specialists. Also, since the main focus of the series was to emphasize the role of nailfold capillaroscopy at the time of diagnosis, we did not include follow-up data in this report.

## Conclusion

Capillaroscopy is a quick and noninvasive tool that is useful in the diagnosis and management of myositis, especially in the absence of Raynaud’s phenomenon and while waiting for confirmatory serologies, imaging, and biopsy results. In clinics and in areas where NVC is not accessible, nailfold evaluation with a dermatoscope is a cheap, accessible, and reliable alternative to identify normal or abnormal patterns, which can in turn influence diagnostic investigations and management. NVC in IIM may be especially useful in cases with atypical presentations when patients may be initially assessed by non-rheumatologists. We encourage other specialists who are likely to see patients with IIM, including internists, dermatologists, neurologists, and pulmonologists, to include NVC as part of their initial diagnostic work-up.
